# The Value of Open Spaces in Great Cities

**Published:** 1913-05-10

**Authors:** John Fletcher Little

**Affiliations:** Park Crescent, W.


*To the Editor of* The Hospital.

### The Value of Open Spaces in Great Cities.

*To the Editor of* The Hospital.

Sir,—I wish to draw the attention of the profession, and especially of the sanitary branch, to what is going on at present in Regent's Park, London, and which, if not stopped, will lead to buildings being erected over one-fourth of the extent of the Park.

There are a number of enclosures, in the aggregate amounting to one hundred acres, in each of which a modest villa stands, interfering little with the amenities or the sanitary value of this great open space. One of these enclosures, consisting of eight or nine acres, close to the Botanic Gardens in the centre of the Park, has been leased to the Bedford College for Women, and six enormous buildings have been erected, which constitute a great encroachment on the sanitary value of the Park. There is immediate danger that the remaining enclosures will be dealt with similarly.

Sanitarians are agreed as to the immense value of open spaces in cities and towns, and I ask for the support of the medical press and profession, and of the medical officers of health, sanitary associations, and all interested in the maintenance and improvement of the public health.

A non-political meeting of protest against this reactionary step will be held in the Park, near the Band-stand, at 3 o'clock on the afternoon of Sunday, May 18.—Yours faithfully,

John Fletcher Little.

Park Crescent, W., April 1913.

